# The impact of a needs-oriented dental prophylaxis program on bacteremia after toothbrushing and systemic inflammation in children, adolescents, and young adults with chronic kidney disease

**DOI:** 10.1007/s00467-021-05153-1

**Published:** 2021-07-23

**Authors:** Karolin Höfer, Anna Turnowsky, Rasmus Ehren, Christina Taylan, Georg Plum, Hanna Witte, Michael J. Noack, Lutz T. Weber

**Affiliations:** 1 Department of Operative Dentistry and Periodontology, Center of Dental Medicine, University of Cologne, Kerpener Strasse 32, D-50931 Cologne, Germany; 2 Pediatric Nephrology, Department of Pediatrics, Faculty of Medicine, and University Hospital Cologne, University of Cologne, Cologne, Germany; 3 Institute for Medical Microbiology, Immunology and Hygiene, University Hospital of Cologne, Cologne, Germany

**Keywords:** Dental prophylaxis, Bacteremia, Inflammation, Toothbrushing, Renal insufficiency, Chronic, Oral health

## Abstract

**Background:**

Chronic kidney disease (CKD) still leads to high mortality rates, mainly due to cardiovascular disease. One important influencing factor is persisting low-grade chronic inflammation partly maintained by gingivitis that favors transient bacteremia during daily activities such as toothbrushing.

**Methods:**

To examine whether intensive dental prophylaxis can restore oral health, reduce the prevalence of bacteremia and degree of systemic inflammation indicated by CRP levels, we conducted this pilot study examining 30 CKD patients aged 6–26 years, 15 receiving intensive prophylaxis (IP), 15 receiving treatment as usual (TAU) serving as control group. There were three appointments for examination, each 10 ± 1 weeks apart (at baseline, after intervention periods one and two, when TAU also received IP, and the IP group stopped prophylaxis).

**Results:**

The gingival index (GI) in the IP group decreased by 90% (GI 0.09; p=0.001), resulting in almost healthy gingiva. There was no significant change in CRP or prevalence of bacteremia. General prevalence of bacteremia after toothbrushing was 9.5% affecting 7 (26%) of the participants. In three participants, bacteremia dissolved after IP, in one after TAU. Two patients developed bacteremia ≥ 10 weeks after ending IP. We identified eight different bacterial species.

**Conclusions:**

We were able to show that IP can effectively treat gingivitis. It might be a promising approach to reduce systemic inflammation and subsequently lower premature cardiovascular disease, despite the lack of statistical significance. Future research requires a larger patient cohort to enable matched treatment groups with long-term follow-up and molecular detection methods for bacteremia.

**Graphical abstract:**

**Figure:**
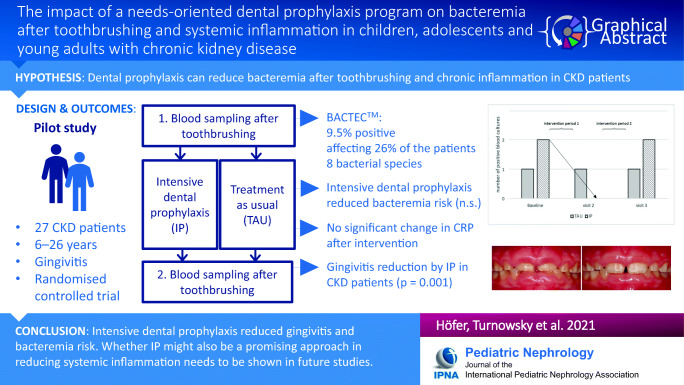
A higher resolution version of the Graphical abstract is available as Supplementary information

**Supplementary Information:**

The online version contains supplementary material available at 10.1007/s00467-021-05153-1.

## Introduction

Despite extensive progress in the treatment of patients affected by chronic kidney disease (CKD), morbidity and mortality still remain unacceptably high [1]. One of the major causes of death is cardiovascular diseases (CVD) [2, 3].

Highest mortality rates in patients on chronic dialysis have been linked to increased inflammatory blood markers for more than 20 years now [4]. In past research concerning the most reliable biomarkers to predict cardiovascular outcome and mortality in CKD patients, the best evidence has been found for C-reactive protein (CRP) and interleukin-6 (IL-6) [5]. There is evidence that elevated CRP levels ≥ 5 mg/l are strongly associated with calcification of coronary arteries and aorta, which are already present in up to 92% of young adults with childhood-onset chronic kidney failure [6].

Among other factors, poor oral health in patients suffering from CKD is one important, yet often underestimated, source of chronic inflammation [7, 8]. Examining the oral condition in patients with CKD, studies demonstrate there is a much higher prevalence of gingivitis, plaque accumulation, attachment loss, enamel hypoplasia, and gingival bleeding or gingival hyperplasia compared to systemically healthy controls whereas caries prevalence remains low, even when kidney function decreases [9, 10].

There is strong evidence that oral bacteria pass to the patients’ blood not only during invasive dental procedures such as tooth extractions, but also due to daily routine activities such as toothbrushing or flossing, with a bacteremia risk of up to 78% [11]. Children requiring conservative dental restorations show 38.5% bacteraemia after toothbrushing. Viridans streptococci, part of the oral cavity and playing a role in the development of infective endocarditis, could be detected in more than 50% of the samples [12]. Within 15 min after dental intervention, the immune system of healthy patients eliminates the bacteraemia [13].

Bacteria can enter the bloodstream through different pathways. They can enter via periapical lesions via the root canal or through the transitional epithelium passing the gingival tissue. Therefore, it seems plausible that poor oral health (e.g., gingivitis) and trauma during dental intervention correlates with the risk for bacterial transfer [14].

In addition to increasing the risk for bacterial translocation, poor oral health also contributes to the development and preservation of chronic systemic inflammation. In their meta-analysis, Paraskevas et al. stated that patients with periodontitis showed an increase in CRP by an average of 1.6 mg/l [14]. There are further studies demonstrating the correlation between poor oral health and increased intima media thickness as an example of the link between oral and cardiovascular pathology [15, 16].

Taking into account all these factors, the development of interventional studies to examine the effect of restoring oral health on systemic inflammation is necessary. Some argue that non-surgical dental intervention can restore oral health but has no significant impact on systemic inflammation [17, 18] while others observe a decrease in high sensitivity CRP (hsCRP) [19, 20]. Tonetti et al. demonstrated that intensive periodontal care initially results in an increase of proinflammatory biomarkers such as CRP and IL-6 combined with a decline in endothelial function, but that there is a significant positive impact on these factors 60 days after treatment. They state that there is a positive correlation between the degree of improvement in oral health with the improvement of inflammatory condition and endothelial function, so dental care becomes a promising approach in improving long-term morbidity and mortality [21].

We hypothesized that an adequate demand-based dental prophylaxis could not only restore poor oral health in patients with CKD but also lower the prevalence of transient bacteremia after toothbrushing and proinflammatory biomarkers seen as parameters of systemic microinflammation. Designing a pilot study, the main focus was on feasibility of our approach in a real-world cohort of patients with CKD.

## Methods

### Study design and study group

This interventional pilot study was approved by the Ethics Committee of the Faculty of Medicine, University of Cologne, Germany, and recorded at The German Clinical Trials Register, registration number DRKS00010580. Written informed consent was obtained by the parents/legal guardians and, if appropriate, by the participants themselves.

**Fig. 1:**
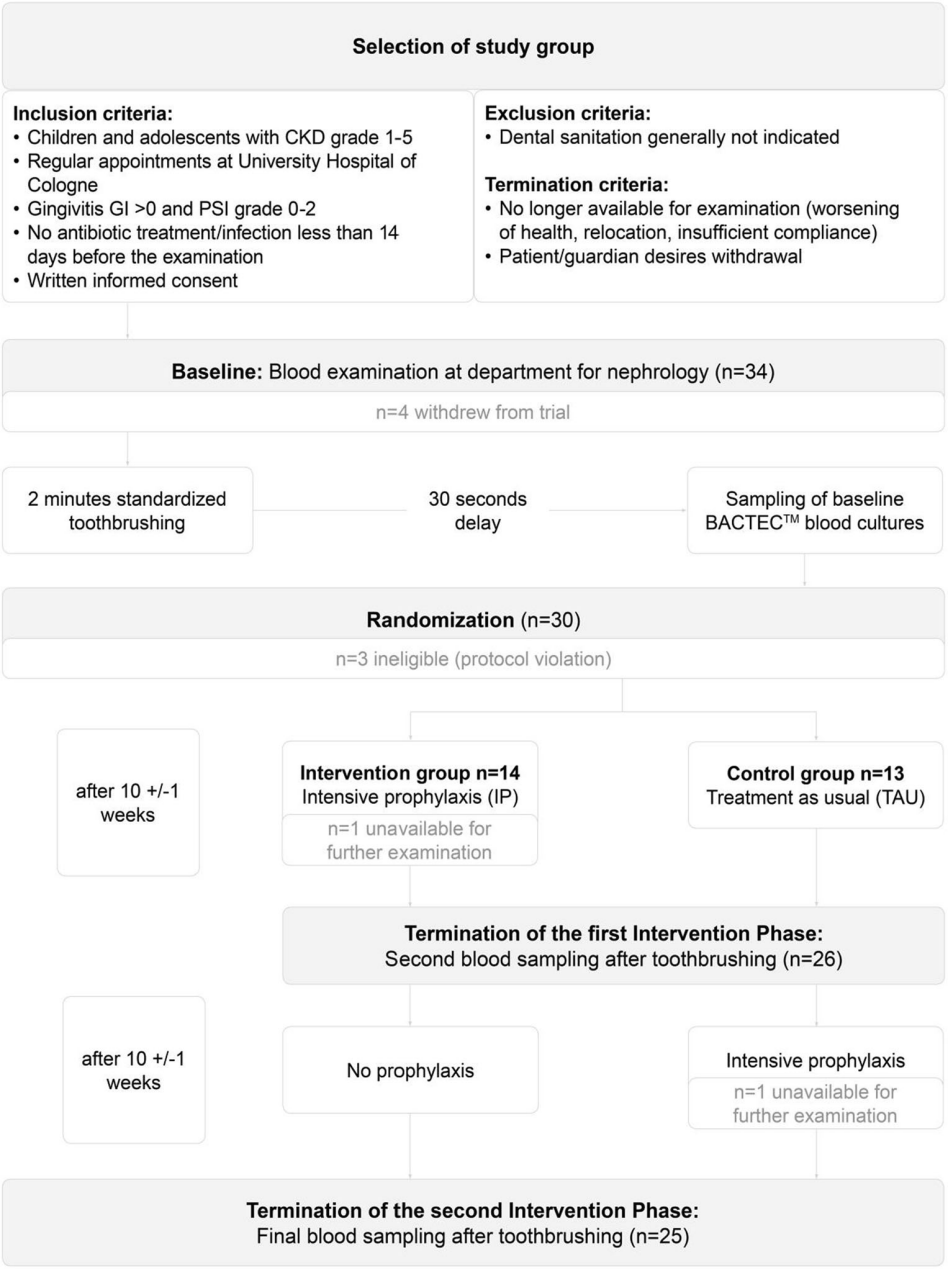
Study design, exploratory randomized controlled trial to evaluate the effect of intensive dental prophylaxis on the prevalence of transient bacteremia after toothbrushing and proinflammatory biomarkers in children, adolescents, and young adults with CKD

We performed a prospective exploratory single-center randomized controlled trial comparing an intensive and demand-related dental prophylaxis program *versus* standard prophylactic dental procedure as currently supported by the German statutory health insurance (Fig. 1). According to the study protocol, the intended treatment period for each patient was 18 to 22 weeks with blood examinations at baseline, 10 ± 1 weeks after randomization (i.e., upon termination of intervention period 1), and another 10 ± 1 weeks later upon termination of intervention period 2.

During the study period from July 2016 to August 2019, we enrolled 34 patients aged 6–26 years at University Hospital of Cologne. We included patients with CKD stages 1–5 according to KDIGO classification [22], conservatively treated as well as already transplanted or dialyzed patients. Any sign of acute infection and/or fever or antibiotic treatment in the last 14 days before participation was an exclusion criterion. Concerning the oral conditions, gingiva index (GI) > 0 and a periodontal screening index (PSI) 0–2 were required. The participants had to be appropriate for dental sanitation. They had the opportunity to stop participating at any time point, if desired. Furthermore, participation ended when the patients were no longer available for examination, e.g., due to worsening of their health status, relocation, or insufficient compliance in realizing appointments.

### Randomization and dental intervention

The clinical exploratory trial consisted of two intervention periods (Fig. 1). The first appointment marked the baseline for every participant: their teeth were professionally brushed for 2 min by one of the investigators; 30 s later, blood was collected for blood culture analysis in aseptic technique and further processed. Afterwards, participants were randomly assigned to either control or intervention group supported by the web-based registration and randomization system TENALEA. To achieve an appropriately random distribution, pseudo-random numbers via computer simulation with permuted 1:1 blocks of variable length assigned patients to intensive prophylaxis (IP) or treatment as usual (TAU) group.

The control group started with one session TAU comparable to their statutory health insurance supply. The intervention group received a needs-oriented step-by-step program for IP that was based on the plaque control program by Axelsson et al. [23]. Determined by their individual need for support, they received weekly appointments at the beginning of the intervention, each of them taking around 30 min, until restoration of oral health. At first, the individual oral situation and need for dental prophylaxis and treatment were evaluated by dental indices such as gingival index (GI), papillary bleeding index (PBI), Quigley-Hein plaque index (QHI), and DMFT/dmft index (*decayed/missing/filled teeth-index, permanent (DMFT), or primary dentition (dmft); gives a measure of oral health status)*. Nutrition counseling concerning cariogenic aspects in close consultation with the nephrological nutritionist and advice on oral hygiene were given. Carious lesions were restored, if necessary. During the second appointment, participants underwent professional dental cleaning (PDC). The third and fourth treatment included PDC and additional prophylaxis measures. Dental indices were reevaluated at every appointment. 10 ± 1 weeks after the baseline visit, the first intervention period terminated with a second blood examination after toothbrushing pursuant to the approach described above. Then, IP group ended the intensive treatment while the participants in TAU group received a singular intensive prophylaxis session: PDC as well as prevention measures. The waiting list control design was used for ethical reasons, so patients in the TAU group were not disadvantaged. Another 10 ± 1 weeks later, the second intervention period terminated with a third blood examination after toothbrushing pursuant to the approach previously described. Apart from the repeatability of measures in TAU group, this examination also served to evaluate the sustainability of the effects of intensive treatment given to IP group during the first intervention period.

### Collection and microbiological analysis of the blood cultures

Participants provided blood samples at three different visits (Fig. 1). For microbiological analysis, we decided to use the BD BACTEC™ Automated Blood Culture System (Becton, Dickinson and Company, Franklin Lakes, NJ) as it is still the standard method for bacterial detection in German hospitals and was already used in previous studies on transient bacteremia [11, 12, 24–26], providing a fast way to detect vital bacteria. Bacteremia as detected by the blood culture bottles was the primary outcome variable.

Patients’ teeth were brushed by the investigator for 120 s using a smear layer of Dentagard© toothpaste (Colgate-Palmolive, New York, NY) and the powered toothbrush Oral-B Professional Care Triumph R© (Procter & Gamble Company, Cincinnati, OH). The brushing technique was standardized, especially regarding the contact pressure and time per quadrant supported by vibration and visual signs given by the Oral-B© toothbrush, to ensure comparability within the patient cohort. Another 30 s after brushing, aseptic blood sampling was performed according to the recommendations of the Robert-Koch-Institute (RKI), Berlin, Germany [27–29]. For ethical reasons, the study-related blood sampling was always linked to an independently necessary blood examination of nephrological indication. After standardized toothbrushing and the delay time of 30 s, 16 ml of blood was taken and immediately split into two samples: 8 ml to inoculate the anaerobic bottle, another 8 ml for the aerobic one. After an incubation time of up to 14 days, the positive cultures indicated by change of fluorescence were further analyzed to identify the organisms.

### CRP and hsCRP

To examine the impact of an intensive oral prophylaxis not only on the oral health of the participants, but also on their chronic inflammatory syndrome, CRP collected from patients’ records at the three different time points ±5 days served as an additional outcome variable.

The collected data contained the commonly measured CRP, detecting elevated levels from 3 mg/l, and for some patients the even more precise hsCRP, allowing for a detection from 0.6 mg/l. A level of ≥ 3 mg/l is sufficient to identify patients with a highly increased cardiovascular risk, whereas inclusion of hsCRP allows conclusions on patients with a potential intermediate or low risk [30].

In addition to CRP/hsCRP, we collected data for the analysis of serum albumin, creatinine and 25-hydroxycholecalciferol (25-OH-vitamin D) at the three different time points to assess changes in nutritional status, kidney function, or calciotropic hormone axis.

### Statistical analysis

The results were analyzed using SPSS statistical software, version 24.0 for Mac OS. For baseline comparison, t-test was used to assess continuous variables controlled for normal distribution by Shapiro–Wilk and Levene’s test (age, GFR, GI). Continuous parametric data was compared by Mann–Whitney-U test (PBI, QHI, DMFT/dmft). In case of binary data (sex, primary disease, therapy, medication), Fisher’s exact test was performed.

To evaluate the prevalence of bacteremia after toothbrushing, between-group differences were assessed for significance by Fisher’s exact test and within-group differences by Cochran’s Q and McNemar tests.

Concerning the CRP level, values were determined as “increased” (CRP ≥ 3 mg/l) or “not increased” (CRP < 3 mg/l). The resulting binary data was treated according to blood culture analysis (Fisher’s exact test, Cochran’s Q test, McNemar test). For further metric analysis, we used the more precise hsCRP values. Values defined as “< 0.6 mg/l” were reduced to a mean value of 0.3 mg/l to prevent the overestimation of inflammation. Values indicated as “< 3 mg/l” were not considered to be sufficiently specific and were excluded from this analysis. Additionally, the difference in hsCRP between visits was calculated. Resulting nonparametric data was analyzed by Mann–Whitney-U test and parametric values by t-test for independent samples. Furthermore, Friedman and Wilcoxon rank-sum tests were performed to detect significant changes within groups. The same methods were used regarding the available data for serum albumin, creatinine, and 25-hydroxy-vitamin D. Statistical significance was set at p < 0.05.

## Results

### Characteristics of the study group

We enrolled 34 patients. Four participants decided to leave before randomization for personal reasons and three patients had to be excluded as they received antibiotic treatment at baseline examination. Their collected data was not considered for statistical analysis. Two of the remaining patients had to be excluded during the study due to insufficient compliance in realizing appointments and the desire to resign. Data of these two patients was considered for final analysis until drop out.

Baseline demographic and clinical characteristics are presented in Table 1. No significant difference between control (TAU) and treatment group (IP) could be found.

**Table 1:** Demographic and clinical characteristics of the control and intensive prophylaxis group

Characteristic	Total (n = 27)	IP (n = 14)	TAU (n = 13)	p-value
*Males*	15 (55.6%)	9 (64.3%)	6 (46.2%)	0.45
Age in years	14.4 ± 5.3	13.8 ± 5	15 ± 5.8	0.56
GFR *in ml/min/1.73 m*^*2*^	35 ± 22	37.9 ± 26.2	31.8 ±16.8	0.48
Primary disease				
*CAKUT*	11 (40.7%)	8 (57.1%)	3 (23.1%)	0.12
*Glomerulopathy*	7 (25.9%)	2 (14.3%)	5 (38.5%)	0.21
*Ciliopathy*	6 (22.2%)	2 (14.3%)	4 (30.8%)	0.39
*Systemic disease*	1 (3.7%)	0	1 (7.7%)	0.48
*Others*	2 (7.4%)	2 (14.3%)	0	0.48
Therapy				
*Conservative*	7 (25.9%)	2 (14.3%)	5 (38.5%)	0.21
*Dialysis*	3 (11.1%)	2 (14.3%)	1 (7.7%)	1.0
*Post-transplant*	15 (55.6%)	9 (64.3%)	6 (46.2%)	0.45
*Post-transplant, dialysis*	2 (7.4%)	1 (7.1%)	1 (7.7%)	1.0
Medication				
*Immunosuppression*	19 (70.4%)	10 (71.4%)	9 (69.2%)	1.0
*—Including cyclosporin*	5 (18.5%)	3 (21.4%)	2 (15.4%)	1.0
*Amlodipine*	16 (59.3%)	8 (57.1%)	8 (61.5%)	1.0
*Vitamin C*	4 (14.8%)	2 (14.3%)	2 (15.4%)	1.0
*Vitamin D*	23 (85.2%)	11 (78.6%)	12 (92.3%)	0.6
DMFT/dmft	0.6 ± 1	0.5 ± 1	0.6 ± 1	0.87
PBI	1.1 ± 0.7	1 ± 0.6	1.1 ± 0.8	0.94
GI	1 ± 0.6	0.9 ± 0.5	1.2 ± 0.8	0.34
QHI	2.5 ± 1	2.7 ± 1	2.3 ± 1	0.19

*IP* intensive prophylaxis, *TAU* treatment as usual, *GFR* glomerular filtration rate, *CAKUT* congenital anomalies of the kidney and urinary tract, *DMFT/dmft* decayed/missing/filled teeth-index, permanent (DMFT), or primary dentition (dmft), *PBI* papillary bleeding index, *GI* Löe-Silness gingival index, *QHI* Quigley-Hein plaque index; Characteristics for categorial variables are presented as n counts and (percentage), and continuous variables as mean and ± SD standard deviation

### Improvement of oral health

**Fig. 2:**
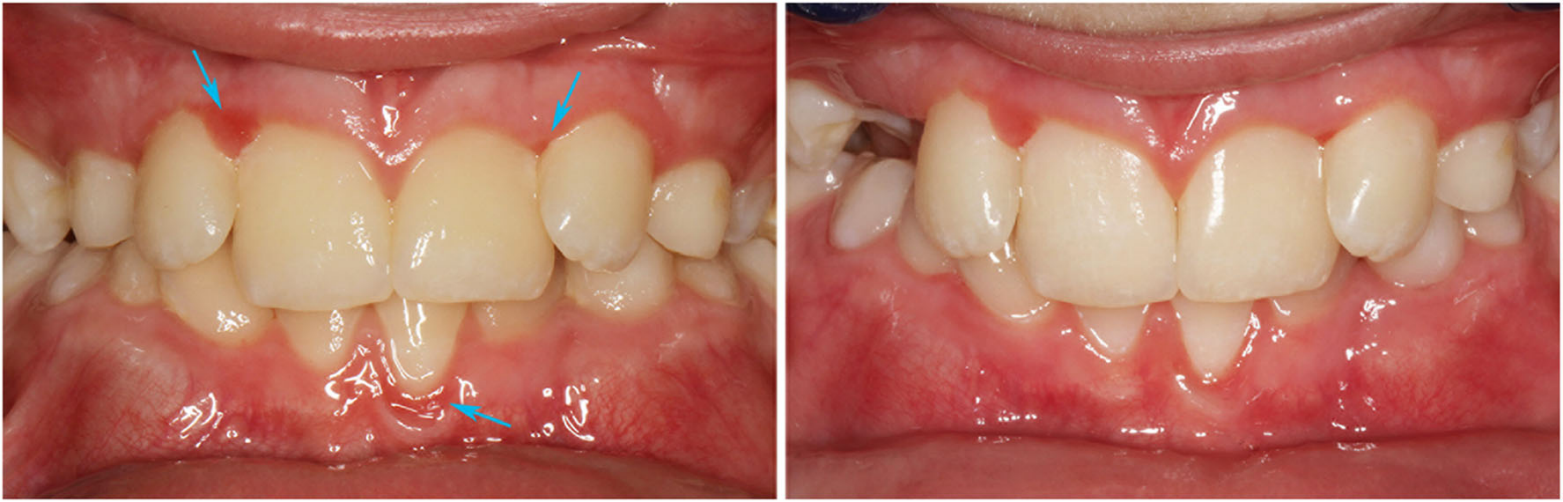
This 10-year-old study patient shows generalized moderate gingivitis, with localized severe inflammation at gingival margin (GI 3). Large plaque accumulations are generally visible on the hard tooth substance. After 3 individual prophylactic sessions, including professional tooth cleaning and motivating cleaning instruction, generalized gingivitis is reduced (GI 0-1). Due to the malpositioned teeth in the upper jaw, there are plaque retention sites that promote the repeated development of gingivitis in the absence of oral hygiene. Additional orthodontic treatment could facilitate oral hygiene

**Fig. 3:**
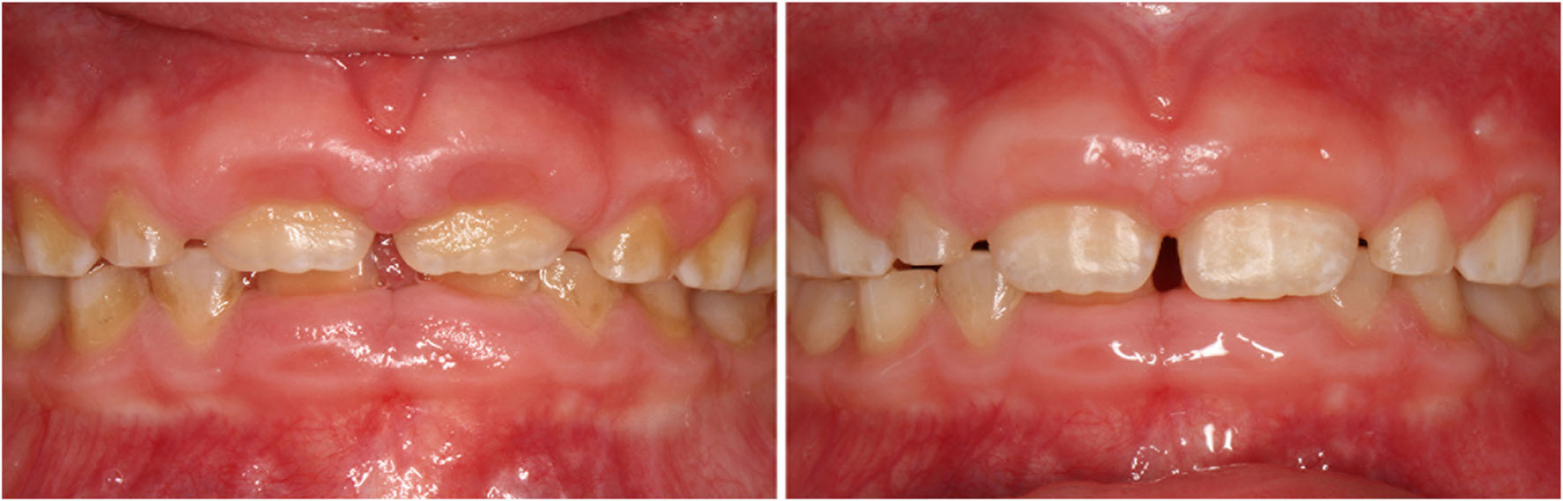
This 6-year-old study patient shows generalized gingivitis (GI 3) with signs of inflammation, local hyperplasia, and increased pocket depth (PD=6mm). The presence of soft and hard tissue illustrates poor oral health condition. The intensive needs-oriented program improved oral hygiene to almost no signs of gingival inflammation in 4 sessions (GI 0). The measuring of the pocket depth showed a reduction by 3 mm based solely on non-surgical treatment

Throughout the study, the needs-oriented prophylaxis program was significantly superior compared to TAU concerning the oral health situation. Figures 2 and 3 serve as examples for common dental health problems in our specific cohort and illustrate the improvements achieved by intensive prophylaxis. The number of dental prophylaxis sessions to regain oral health differed considerably between patients and lay between 2 and 5 sessions, as the number of dental sessions in the IP group was determined individually and according to needs. DMFT/dmft, PBI, GI, and QHI at baseline are given in Table 1. The gingival index (GI) in the IP group decreased by 90% (GI 0.09; SD 0.09; p = 0.001) resulting in an almost healthy gingiva in this group after intensive prophylactic measures. Gingivitis in the TAU group improved by 15% (GI 0.97; SD 0.58). Eleven study patients were in mixed dentition (MD) at baseline examination and the other 16 patients had permanent dentition (PD). The patients with MD had a DMFT/dmft index of 0 for IP and 0.4 for TAU, the patients with PD had a DMFT index with a mean of 0.88 (SD 1.13) for the IP and 0.89 (SD 1.17) for TAU group. Higher DMFT/dmft index means worse oral health.

### Prevalence of bacteremia after toothbrushing

A total of 74 pairs of blood culture bottles were available for analysis. One of the patients showed a highly increased CRP level of 21.1 mg/l with suspected pneumonia at his first visit, we excluded the associated blood culture from further analysis although it revealed a skin germ (*Staphylococcus hominis*). Seven additional blood cultures contained viable bacteria indicated by change of fluorescence in seven different participants. We were able to identify eight different bacterial species. The detection rate of overall transient bacteremia after toothbrushing in patients with CKD was 9.5%. Twenty-six percent of the participants (n = 7) developed bacteremia at one of their three visits (Table 2).

**Table 2:** Identification of bacterial species in positive BACTEC^TM^ culture bottles 30s after toothbrushing

Treatment group	Number	Blood culture 1^a^	Blood culture 2^b^	Blood culture 3^c^
TAU	1	neg.	*Staphylococcus epidermidis*	neg.
	2	*Streptococcus mitis, Neisseria flavescens*	neg.	neg.
	3	neg.	neg.	*Propionibacterium acnes*
IP	4	neg.	neg.	*Staphylococcus epidermidis, Propionibacterium acnes*
	5	neg.	neg.	*Staphylococcus hominis*
	6	*Streptococcus constellatus, Gemella morbillorum*	neg.	neg.
	7	*Actinomyces oris*	neg.	neg.

^a^Blood culture at baseline before any treatment, ^b^Blood culture after intervention period 1, when TAU received prophylactic treatment as usual, and IP received intensive dental prophylaxis, ^c^Blood culture after intervention period 2, when TAU underwent intensive dental prophylaxis, and IP received no prophylaxis

**Fig. 4:**
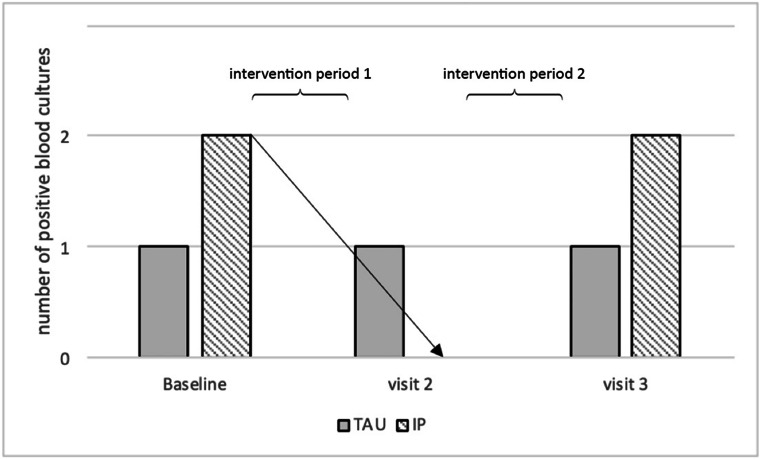
Number of positive BACTEC blood culture bottles 30s after toothbrushing at baseline, after intervention periods 1 and 2 separately for TAU and IP. After intensive dental prophylactic measures in IP group, bacteremia was reduced from 2 to 0 positive blood cultures (signaled by arrow, p = 0.368)

In three participants, bacteremia dissolved after intensive treatment. The same occurred in one patient after TAU. Two patients showed bacteremia ≥ 10 weeks after ending IP. One patient in the TAU group showed bacteremia only at the third visit after having received the intensive measures during intervention period 2. Regarding the results in the IP group, the number of positive BACTEC^TM^ culture bottles decreased after intensive prophylactic treatment (p = 0.368, Fig. 4). Comparison between the groups revealed no significant differences (p = 1.0 at the three visits).

### Change in CRP, serum albumin, creatinine, and 25-OH-vitamin D

Analyzing the development of CRP and hsCRP levels during the study period, there was no statistically significant change, neither within the treatment or control group nor in comparison between them. Again, the patient showing CRP 21.1 mg/l was excluded from this analysis as his CRP was rather linked to the beginning pneumonia instead of mirroring chronic inflammation.

At baseline, CRP ≥ 3 mg/l was observed in three patients (2 TAU, 1 IP). CRP values were available for 21/26 patients. 14.3% of the measured values were in the range of high cardiovascular risk evaluated by CRP [30]. After the first intervention period, 1/17 (5.9%) recorded patients demonstrated CRP ≥ 3 mg/l. Also, 1/17 patients (5.9%) showed an increased CRP level after the second intervention period. They both belonged to the IP group. In binary analysis of CRP levels, there was no change by intensive dental care.

The subgroup analysis of hsCRP values shows similar results. Comparison before and after intensive treatment showed a mean decrease of −0.2 ± 0.6 mg/l in IP group (n = 5, max. decrease −1.3, max. increase +0.1, p = 0.655). Even though we detected the highest hsCRP at baseline decreasing throughout the study, the reduction is not statistically significant.

The laboratory parameters serum albumin, creatinine, and 25-OH-vitamin D as markers for nutritional status, kidney function, and calciotropic hormone axis were not significantly influenced by intensive dental prophylaxis (Table 3).

**Table 3:** Impact of intensive dental prophylaxis on CRP, serum albumin, creatinine, and 25-OH-vitamin D levels in patients with CKD

Markers	Follow-up		
	Baseline	After first intervention period^1^	After second intervention period^2^
CRP, mg/l			
TAU	2 ± 2.2 (n = 9)	0.3 ± 0 (n = 7)	0.7 ± 1.1 (n = 6)
IP	1.2 ± 0.9 (n = 8)	0.9 ± 1.1 (n = 6)	1.2 ± 1.1(n = 10)
p-value between groups	0.815	0.138	0.368
Serum albumin, g/l			
TAU	44.4 ± 3.1 (n = 11)	45 ± 2.7 (n = 10)	45.3 ± 2.2 (n = 9)
IP	44.7 ± 2.4 (n = 12)	44.8 ± 1.8 (n = 11)	45.4 ± 2.8 (n = 11)
p-value between groups	0.695	0.856	0.979
Creatinine, mg/dl			
TAU	2.8 ± 2.7 (n = 12)	2 ± 0.9 (n = 11)	2.8 ± 2.9 (n = 12)
IP	2.5 ± 3 (n = 12)	1.5 ± 0.7 (n = 11)	1.6 ± 0.8 (n = 12)
p-value between groups	0.347	0.193	0.198
25-OH-vitamin D, μg/l			
TAU	34.8 ± 7.9 (n = 8)	46.7 ± 6.6* (n = 7)	47.3 ± 4.8 (n = 3)
IP	40.3 ± 14.7 (n = 8)	44.8 ± 10.3 (n = 8)	38.8 ± 14 (n = 9)
p-value between groups	0.370	0.685	0.341

^1^During the first intervention period, IP received intensive treatment; ^2^During the second intervention period, TAU received intensive treatment; *TAU* treatment as usual group, *IP* intensive prophylaxis group, *p = 0.046 compared to baseline

## Discussion

The present pilot study was not able to detect a statistically significant change in the prevalence of transient bacteremia by using BACTEC^TM^ blood culture bottles after toothbrushing in patients with CKD following intensive dental prophylactic treatment. In addition, there is no proof for a positive impact on CRP levels as a marker for chronic inflammation. The gingival index (GI) in the IP group decreased by 90% (GI 0.09; SD 0.09; p = 0.001), resulting in an almost healthy gingiva in this group after intensive prophylactic measures. Gingivitis in the TAU group improved by only 15% (GI 0.97; SD 0.58), proving superiority of IP concerning oral health.

Our study resulted in a detection of bacteria after toothbrushing in 9.5% of the analyzed samples, affecting 26% of the participants throughout the study period. In addition, two of the three participants who had to be excluded from analysis due to antibiotic prophylaxis showed transient bacteremia throughout the study period, as well. Ensuring this inclusion criterion was rather difficult in our specific cohort and led to several postponements, as patients with CKD often suffer from infections or require antibiotic prophylaxis due to their primary diseases, especially after transplantation or in advanced stages of CKD. In general, the efficacy of antibiotic prophylaxis prior to dental intervention is a controversial topic [31], but should be considered for the development of interventional trials in this field.

The identified organisms in this study are consistent with the work of former investigations [12, 13]. Comparing the prevalence, it has to be considered that most reports, among them the studies by Roberts et al., do not discuss the origin of the identified bacteria like *Staphylococcus epidermidis* or *Staphylococcus hominis* that are likely to be contaminants. This might result in an overestimation of the true prevalence of transient bacteremia in these studies [13]. Two of our isolates (20%, *Streptococcus mitis* and *Streptococcus constellatus*) belong to the large group of Viridans streptococci. Viridans streptococci represent the most frequently isolated group of bacteria with oral origin, resulting in a detection rate of up to 58% after dental procedures in children [12]. *Propionibacterium acnes* was detected in two blood cultures. Analogous to the interpretation by Hartzell et al. [24], contamination by skin bacteria seems highly probable despite the aseptic blood sampling technique that was performed according to the recommendations of the RKI [27–29]. It can be speculated that the diversion of the first ml of each specimen would have further reduced the risk for contamination [32]. Even though a definite conclusion is not possible, most of the bacteria identified in the present study can also be found in the oral flora, supporting the assumption that those cases show indeed true transient bacteremia after toothbrushing [33, 34].

Concerning the detection method, sensitivity is the main issue. Blood culture bottles are the most commonly used medium [11, 12, 24–26], remain the gold standard in clinical practice in German hospitals, and have been used in the preceding project to our study [35]. A more sensitive methods is lysis-filtration, being more time-consuming but allowing not only a more sensitive detection but also information about the bacterial load [36]. Furthermore, authors like Kinane et al. [37] found PCR-analysis to be more sensitive than culture methods (bacteria detection after toothbrushing in 13% vs. 3%) and Marín et al. [38] compared further molecular techniques like direct anaerobic culturing (DAC), lysis-centrifugation (LC), and quantitative PCR-analysis with detection via BACTEC^TM^ cultures. There is so far no generally accepted consensus on the optimal method to be used.

Another important potential influencing factor on the prevalence of bacteremia is the oral health status. To evaluate the oral situation of our participants, we used dental indices like dmft/DMFT, PBI, GI, and QHI (Table 1). Mean dmft/DMFT index was 0.6 ± 1 (median 0, range 0–3) similar to the very low prevalence of dental caries in German 12-year-olds (mean DMFT 0.7 [39]). In a preceding project [35], children with congenital heart diseases showed a higher prevalence of bacteremia after toothbrushing (21.4%) and also a greater caries prevalence with a mean DMFT of 4.6. Patients with CKD show lower caries prevalence, e.g., due to the alkaline oral pH that protects against acidogenic microflora associated with caries [40]. Whether or not caries influences the trespassing of bacteria into the blood stream is yet to be determined.

Mean PBI at baseline was 1.1 ± 0.7 (median 0.9, range 0.2–3.0) equivalent to a rather mild gingival inflammation [41]. Appropriately, GI according to Löe and Silness [42] was 1 ± 0.6 (median 1.0, range 0.2–3.0) at baseline. In addition, our participants demonstrated mild to moderate plaque accumulation as QHI [43] was 2.5 ± 1 (median 2.4, range 1.3–5.2) at baseline.

Gingivitis is one of the frequently reported soft tissue manifestation of CKD and is confirmed by the present baseline examinations of the dental indices. The increased plaque levels and gingivitis indices at baseline demonstrate inadequately performed oral hygiene measures prior to dental instruction and prophylaxis measures. Even in high-risk transplant patients, a demand-oriented preventive dental service can lead to an improvement in oral hygiene at home and thus improve the local inflammatory process in the oral cavity [40].

Concerning the relationship between oral health and risk for transient bacteremia, Silver et al. [44] described an increase from 16% prevalence in patients with a GI 0–0.75 to 68% in patients with moderately to severely inflamed gingiva (GI 2.26–3). Other investigators failed to prove a statistically significant impact of oral health on the trespassing of oral bacteria into the bloodstream [25, 45]. Nevertheless, Maharaj et al. [25] describe a difference in detection from 8% in GI 0.1–2.0 up to 16% in GI 2.1–3.0 and also an increase to 19% in patients with severe plaque accumulation which might be of clinical significance.

Regarding the mild to moderate gingival inflammation and plaque accumulation at baseline and even significantly lower indices after intensive treatment, the reported low prevalence of bacteremia is in line with former work on patients with similar oral conditions. Nevertheless, the given dental indices have to be interpreted with caution as they are calculated on the basis of averages and disguise the locally high plaque-induced gingivitis seen in our participants.

Another potential contributing factor to the prevalence of bacteremia is the grade of invasion caused by dental intervention [31]. In a study by Lockhart et al. [26], toothbrushing provided a 32% risk of developing bacteremia whereas tooth extraction without antibiotic prophylaxis provoked bacteremia in 80% of the cases. This finding is confirmed by other investigators such as Roberts et al. [12]. Even the difference between manual and electric toothbrushing is significant (48% vs. 78%), probably due to the more vigorous brushing with electric devices [11]. In the present study, we chose powered toothbrushing performed by three different investigators. Patients participating in our project were fully awake during the toothbrushing compared to several other studies where brushing was performed under general anesthesia prior to other dental interventions [11, 12]. Most studies on conscious participants have examined adults. This circumstance might have had an impact on the vigorousness and thoroughness during the toothbrushing and in consequence might be one of the reasons for the relatively low prevalence of bacteremia in our study. Furthermore, the conduct under general anesthesia facilitates the blood sampling itself as the vascular access is often easily available and the time interval of 30s between toothbrushing and blood sampling can be fulfilled more strictly.

Apart from transient bacteremia detected by blood culture, we also examined laboratory parameters, especially the CRP values of our participants, as markers for the chronic inflammation that constitutes one of the main factors for cardiovascular risk and subsequently for mortality. The fact that there is a significant association between gingivitis and chronic inflammation even in systemically healthy 15-year olds [46] emphasizes the need for interventional trials already in mild stages of oral inflammation. hsCRP values ranged from 0.2 to 8.4 mg/l with a mean value of 0.6 ± 0.9 mg/l [46] and were substantially lower than in our patient cohort (1.6 ± 1.7 mg/l, range 0.3–5.8 mg/l), which accentuates the special need for action in patients with CKD and gingivitis.

Referring to studies about the impact of non-surgical dental intervention in patients on systemic proinflammatory biomarkers such as CRP, data are scarce. Freitas et al. [19] showed a mean decrease of –0.2 mg/l in their meta-analysis. Fang et al. [47] examined 97 patients with stage 5 CKD and found a significant decrease in hsCRP 3 (–0.3 mg/l, p = 0.005) and 6 months (–0.4 mg/l, p < 0.001) after non-surgical periodontal therapy compared to the control group without treatment. Further studies on adults with CKD confirmed a possible link between periodontal therapy with improvements in oral health and decrease of hsCRP, increase in serum albumin and hemoglobin levels by improving erythropoietin responsiveness [48, 49]. In contrast, Wehmeyer et al. [18] failed to detect a significant improvement in serum albumin or hsIL-6 values, probably attributable to their study design and heterogeneous randomization, especially concerning participants affected by diabetes. Due to heterogeneity concerning the degree of CKD, severity of periodontal disease, periodontal treatment, and chosen outcome, comparison and final conclusion concerning the effect of dental intervention on inflammation and kidney function in CKD patients remain incomplete [19]. In our study, members of the IP group showed a statistically nonsignificant decrease in hsCRP by −0.2 ± 0.6 mg/l (available data n = 5) after receiving intensive prophylactic measures during the first intervention period. The decrease in the TAU group after receiving standard measures appears to be greater (−2.2 ± 2.5 mg/l, n = 6) due to the fact that the baseline value contains two outliers with singularly elevated CRP values (5.8 mg/l and 5.5 mg/l) without clinical signs of infection. Reasons for the elevation remain uncertain, but the singularity of the measurements is an argument for an acute event rather than mirroring chronic microinflammation. As recurrent infections are frequent in patients with CKD, interpreting changes in CRP is challenging.

There were also no significant changes in serum albumin, creatinine, and 25-OH-vitamin D associated with intensive dental care. The significant increase of 25-OH-vitamin D after TAU (mean change +9.8 ± 8.6 μg/l, p = 0.046) might also be attributable to changes in vitamin supplementation, kidney function with impact on the calciotropic hormone axis, or seasonal changes. All of our results have to be interpreted with caution given the limited sample size and data availability due to the exploratory character of the trial.

During 3 years of patient acquisition, several problems became obvious. We were not able to comply with the set interval of 10 ± 1 weeks as given by the study protocol in all of the cases. The mean interval between the visits was 122 ± 67 days (first intervention period) and 174 ± 101 days (second intervention period). The main reasons for this deviation were fluctuation in health as well as logistical difficulties on the part of the participants, especially given the additional effort required by an interdisciplinary clinical trial. Furthermore, not all laboratory parameters were available in all patients, resulting in a discontinuous monitoring throughout the study impeding conclusive evaluation.

Despite the fact that we were not able to show a statistically significant positive influence of intensive dental prophylaxis on transient bacteremia and chronic inflammation, there are implications that intensified dental treatment as applied by our study protocol does not only restore oral health but might also have a systemic positive impact. TAU as supported by the statutory health insurance is not sufficient to preserve or restore oral health in this cohort of chronically ill patients. It seems obvious to deduce that preventing periodontitis by early prophylaxis and improvement of dental care in this population is crucial, especially regarding the presence of chronic inflammation already in young age. Our trial can serve as a helpful pilot for further studies as it depicts valuable information on which potential pitfalls should be considered. Future analyses should treat a larger patient cohort with long-term follow-up and ensure continuous monitoring of laboratory parameters throughout the study period combined with bacteria detection by molecular methods. Implementing a needs-oriented intensive dental prophylaxis with regular appointments is a necessary approach in improving oral health and may also be a promising strategy concerning improvements in general health, systemic inflammation, and premature CVD.

## Supplementary Information

Graphical abstract (PPTX 425 kb)

ESM 1 (DOCX 16.2 kb)
