# Supplementary material for: The impact of a needs-oriented dental prophylaxis program on bacteremia after toothbrushing and systemic inflammation in children, adolescents, and young adults with chronic kidney disease
Source: Pediatr Nephrol. 2021 Jul 23;37(2):403–14. doi: 10.1007/s00467-021-05153-1 (PMC8816805; doi:10.1007/s00467-021-05153-1)
Supplement: Supplementary file 1 — (PPTX 425 kb) [file 467_2021_5153_MOESM1_ESM.pptx]

## Slide 1
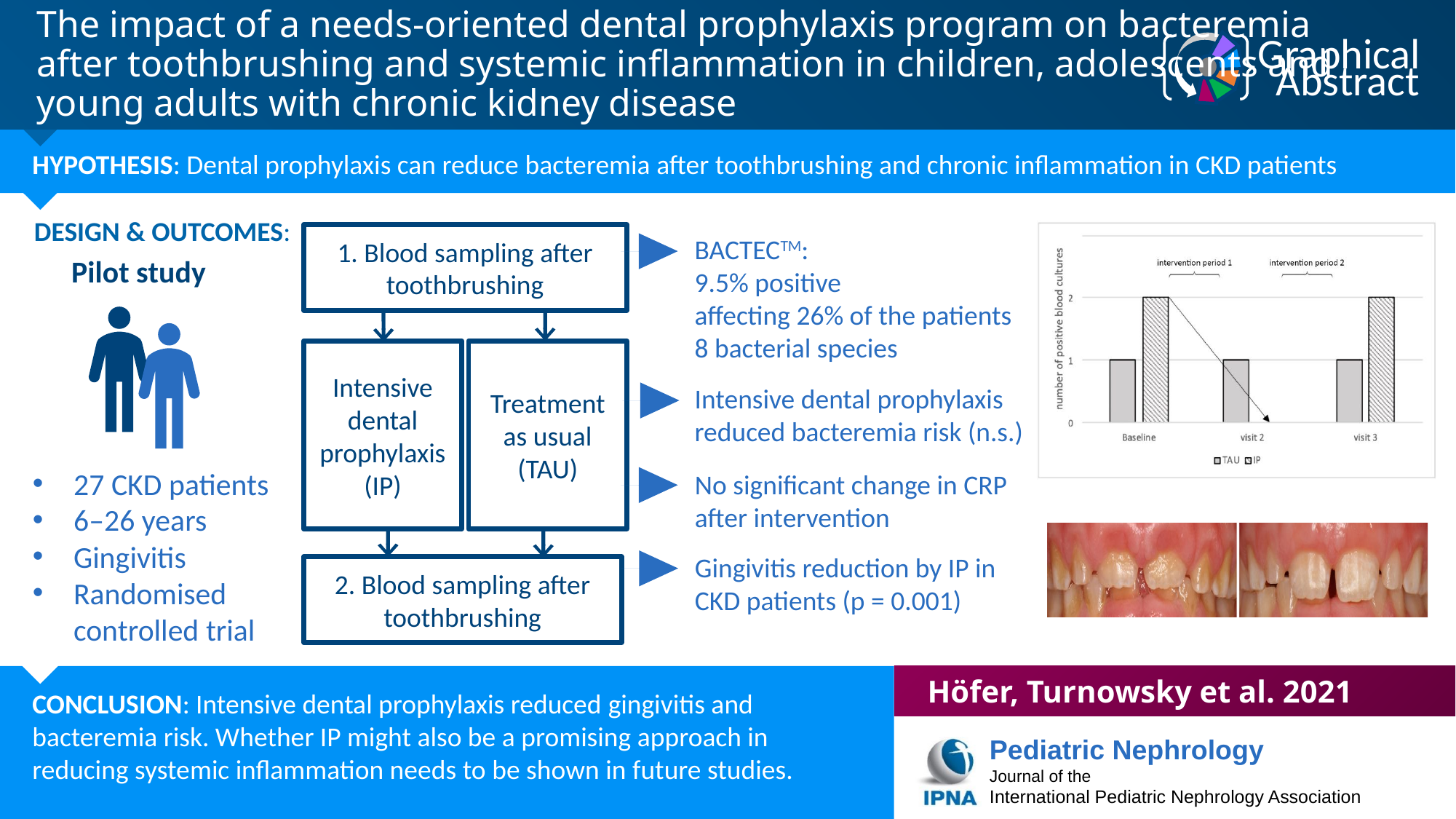

The impact of a needs-oriented dental prophylaxis program on bacteremia
after toothbrushing and systemic inflammation in children, adolescents and
young adults with chronic kidney disease
HYPOTHESIS: Dental prophylaxis can reduce bacteremia after toothbrushing and chronic inflammation in CKD patients
DESIGN & OUTCOMES:
1. Blood sampling after toothbrushing
BACTECTM:
9.5% positive
affecting 26% of the patients 8 bacterial species
Pilot study
Intensive dental prophylaxis (IP)
Treatment as usual (TAU)
Intensive dental prophylaxis reduced bacteremia risk (n.s.)
27 CKD patients
6–26 years
Gingivitis
Randomised controlled trial
No significant change in CRP after intervention
Gingivitis reduction by IP in CKD patients (p = 0.001)
2. Blood sampling after toothbrushing
Höfer, Turnowsky et al. 2021
CONCLUSION: Intensive dental prophylaxis reduced gingivitis and bacteremia risk. Whether IP might also be a promising approach in reducing systemic inflammation needs to be shown in future studies.
