# Supplementary material for: The impact of a needs-oriented dental prophylaxis program on bacteremia after toothbrushing and systemic inflammation in children, adolescents, and young adults with chronic kidney disease
Source: Pediatr Nephrol. 2021 Jul 23;37(2):403–14. doi: 10.1007/s00467-021-05153-1 (PMC8816805; doi:10.1007/s00467-021-05153-1)
Supplement: Supplementary file 2 — (DOCX 16.2 kb) [file 467_2021_5153_MOESM2_ESM.docx]

| **Tab. 4** Age distribution | | | |
| --- | --- | --- | --- |
| Age in years | number of patients  (n=27) | IP  (n=14) | TAU  (n=13) |
| ≥6 to <7 | 1 | 1 | 0 |
| ≥7 to <8 | 3 | 1 | 2 |
| ≥8 to <9 | 1 | 1 | 0 |
| ≥9 to <10 | 2 | 0 | 2 |
| ≥10 to <11 | 1 | 1 | 0 |
| ≥11 to <12 | 2 | 2 | 0 |
| ≥12 to <13 | 1 | 0 | 1 |
| ≥13 to <14 | 0 | 0 | 0 |
| ≥14 to <15 | 1 | 1 | 0 |
| ≥15 to <16 | 2 | 1 | 1 |
| ≥16 to <17 | 1 | 1 | 0 |
| ≥17 to <18 | 3 | 1 | 2 |
| ≥18 to <19 | 2 | 1 | 1 |
| ≥19 to <20 | 3 | 1 | 2 |
| ≥20 to <21 | 2 | 1 | 1 |
| ≥21 to <22 | 1 | 1 | 0 |
| ≥22 to <23 | 0 | 0 | 0 |
| ≥23 to <24 | 0 | 0 | 0 |
| ≥24 to <25 | 0 | 0 | 0 |
| ≥25 to <26 | 0 | 0 | 0 |
| ≥26 to <27 | 1 | 0 | 1 |
